# A novel approach based on preference-based index for interval bilevel linear programming problem

**DOI:** 10.1186/s13660-017-1384-1

**Published:** 2017-05-15

**Authors:** Aihong Ren, Yuping Wang, Xingsi Xue

**Affiliations:** 1 Department of Mathematics, Baoji University of Arts and Sciences, Baoji, 721013 China; 2 School of Computer Science and Technology, Xidian University, Xi’an, 710071 China; 3 College of Information Science and Engineering, Fujian Provincial Key Laboratory of Big Data Mining and Applications, Fujian University of Technology, Fuzhou, 350118 China

**Keywords:** bilevel programming, interval number, preference-based index, estimation of distribution algorithm

## Abstract

This paper proposes a new methodology for solving the interval bilevel linear programming problem in which all coefficients of both objective functions and constraints are considered as interval numbers. In order to keep as much uncertainty of the original constraint region as possible, the original problem is first converted into an interval bilevel programming problem with interval coefficients in both objective functions only through normal variation of interval number and chance-constrained programming. With the consideration of different preferences of different decision makers, the concept of the preference level that the interval objective function is preferred to a target interval is defined based on the preference-based index. Then a preference-based deterministic bilevel programming problem is constructed in terms of the preference level and the order relation $\preceq_{mw}$. Furthermore, the concept of a preference *δ*-optimal solution is given. Subsequently, the constructed deterministic nonlinear bilevel problem is solved with the help of estimation of distribution algorithm. Finally, several numerical examples are provided to demonstrate the effectiveness of the proposed approach.

## Introduction

The bilevel programming problem is a hierarchical optimization problem involving decision processes with two decision makers, the so-called leader or upper level decision maker and the so-called follower or lower level decision maker. In such a hierarchical decision framework, the leader first specifies a strategy, and then the follower chooses a strategy in view of the leader’s decision. Two decision makers attempt to optimize their respective objective functions, but are affected by the actions with each other. In the past few decades, the bilevel programming problem has widely applied in numerous areas including transport network design [1, 2], price control [3], principal-agent problems [4, 5], supply chain management [6, 7], engineering design [8, 9], electricity markets [10]. Nevertheless, the bilevel programming problem is generally non-convex and non-differentiable, and it is NP-hard [11] and quite difficult to deal with. Many researchers have worked on this topic, like Dempe [12], Colson *et al.* [13], Kalashnikov *et al.* [14], Bard [15], Dempe [16], Dempe *et al.* [17] and Zhang *et al.* [18].

It is well known that, in the conventional bilevel programming problem, the coefficients in both objective functions and constraints are deterministic or crisp. However, in practice, we may be faced with some situations where the coefficients in the problem are uncertain. To tackle these uncertain coefficients, stochastic and fuzzy approaches are commonly used, and accordingly stochastic and fuzzy bilevel programming problems are proposed. In stochastic bilevel programming problems [19], the coefficients are viewed as random variables with known probability distributions; whereas in fuzzy bilevel programming problems [20, 21], the coefficients are regarded as fuzzy sets with known membership functions. It is worth mentioning that the probability distributions and the membership functions are of great importance to formulate and solve these two classes of problems. However, it is a complicated task for decision makers to choose appropriate probability distributions or membership functions in the case of stochastic or fuzzy uncertainties. To efficiently cope with the uncertainties, there emerges the interval programming, which becomes a prominent tool for tackling decision problems with uncertain parameters and it only requires to calculate the bounds of the uncertain coefficients. On this basis, the interval bilevel programming problem, whose coefficients of both objective functions or constraints are interval numbers, begins to attract more and more researchers, but to the best of our knowledge, academic papers about this kind of problem are still few.

Some studies as regards the interval bilevel linear programming problem exist in the literature. Among them, Abass [22] applied the possibility degree of interval number to reduce interval inequality constraints into deterministic inequality forms and employed the midpoint and radius of interval number to convert interval objective functions of the upper and lower levels into both deterministic objective functions. Then the interval bilevel linear programming problem with interval coefficients in both objective functions and constraints was transformed into a deterministic bilevel optimization problem. In this way, a better optimal solution can be obtained with a smaller possibility degree of the constraint, However, a smaller possibility degree implies a smaller reliability of the uncertain constraint. Meanwhile, this transformation of interval inequality constraints into crisp equivalent forms could lead to the uncertainty of intervals lost to some extent. Wang and Du [23] investigated the bilevel linear programming problem with interval coefficients in both objective functions, proposed a concept of the optimal solution and developed an approach based on a new partial order on intervals to cope with the problem. For the same kind of problem, Calvete and Galé [24] designed two enumerative algorithms to obtain the best and worst optimal solutions of the problem and provided the optimal value range for the upper level objective function. In the above two works, the constraints are confined to crisp inequality forms. Subsequently, Ren and Wang [25] pointed out that the algorithm of finding the worst optimal solution (KBW) proposed by Calvete and Galé [24] was not always yield a correct solution, and presented two types of cutting plane methods to compute the best and worst optimal solutions. Moreover, the two algorithms were extended to determine the best and worst optimal solutions of the general interval bilevel linear programming problem. Nehi and Hamidi [26] also discussed the drawbacks of the KBW algorithm in [24], proposed a revised algorithm (RKBW) and extended the revised algorithm to deal with the general interval bilevel linear case. It should be noted here that the best and worst optimal solutions obtained by these above three studies are two extreme cases, and thus it is difficult to reflect different preferences of different decision makers in practice.

Under the above consideration, from a point of decision makers’ preferences, our objective is to propose an alternative way based on preference-based index to deal with the interval bilevel linear programming problem in which all coefficients in both objective functions and constraints are intervals. To this end, we first convert the original constraint region into its crisp equivalent form by combining normal variation of interval number with chance-constrained programming [27], and thus transform the original problem into an interval bilevel programming problem where interval coefficients are in both objective functions only. This kind of transformation keeps the uncertainty of the original constraint region as much as possible. Taking into account different preferences of different decision makers, the preference level that the interval objective function is preferred to a target interval is defined in terms of preference-based index developed by Ruan *et al.* [28]. Then a preference-based deterministic bilevel programming problem can be constructed by replacing the upper level interval objective function with corresponding preference level and using the order relation $\preceq_{mw}$ to handle the lower level interval objective function. For the constructed nonlinear deterministic problem, we adopt an estimation of distribution algorithm to solve it. Finally, several numerical examples are provided to demonstrate the feasibility of the proposed method.

The remainder of this paper is organized as follows. Section 2 briefly recalls some basic definitions and preliminary results related to interval numbers. In Section 3, we introduce the interval bilevel linear programming problem. In Section 4, a new approach based on preference-based index is proposed to handle the interval bilevel linear programming problem. Section 5 provides some numerical examples to illustrate the proposed method and make some comparisons with the existing methods. Finally, we conclude the paper and suggest directions for future work in Section 6.

## Preliminaries

Let *R* be the set of all real numbers.

An interval number $c^{I}$ can be defined as

$$c^{I}=\bigl[c^{-},c^{+}\bigr]=\bigl\{ c|c^{-}\leq c\leq c^{+}, c\in R\bigr\} , $$

where $c^{-}$ and $c^{+}$ are the lower and upper bounds of $c^{I}$, respectively. If $c^{-}=c^{+}$, then $c^{I}$ is a real number.

In terms of its midpoint and half-width, an interval number $c^{I}$ can also be defined as

$$c^{I}=\bigl\langle m\bigl(c^{I}\bigr),w\bigl(c^{I} \bigr)\bigr\rangle =\bigl\{ c|m\bigl(c^{I}\bigr)-w\bigl(c^{I} \bigr)\leq c \leq m\bigl(c^{I}\bigr)+w\bigl(c^{I}\bigr), c\in R\bigr\} , $$

where $m(c^{I})=\frac{c^{-}+c^{+}}{2}$ and $w(c^{I})=\frac {c^{+}-c^{-}}{2}$ are the midpoint and half-width of interval $c^{I}$, respectively.

For $c^{I}_{1}=[c_{1}^{-},c_{1}^{+}]$, $c^{I}_{2}=[c_{2}^{-},c_{2}^{+}]$ and $c^{I}=[c^{-},c^{+}]$, the arithmetical operations on intervals are defined as follows:

(i) $c^{I}_{1}+c^{I}_{2}=[c_{1}^{-}+c_{2}^{-},c_{1}^{+}+c_{2}^{+}]$;
(ii) $c^{I}_{1}-c^{I}_{2}=[c_{1}^{-}-c_{2}^{+},c_{1}^{+}-c_{2}^{-}]$;
(iii) $kc^{I}= \bigl\{ \scriptsize{ \begin{array}{l@{\quad}l} [kc^{-},kc^{+}], & k\geq0,\\ {} [kc^{+},kc^{-},] & k<0. \end{array}}$

With respect to the more details of interval mathematics, please see Moore [29] and Alefeld and Herzberger [30].

Next we recall two types of interval ranking relations.

### Definition 1

[31]

The order relation $\preceq_{mw}$ between two interval numbers $c^{I}_{1}=[c^{-}_{1},c^{+}_{1}]$ and $c^{I}_{2}=[c^{-}_{2},c^{+}_{2}]$ is defined as:

$c^{I}_{1}\preceq_{mw} c^{I}_{2}$ if $m(c^{I}_{1})\leq m(c^{I}_{2})$ and $w(c^{I}_{1})\leq w(c^{I}_{2})$ for minimization problems,

$c^{I}_{1}\preceq_{mw} c^{I}_{2}$ if $m(c^{I}_{1})\leq m(c^{I}_{2})$ and $w(c^{I}_{1})\geq w(c^{I}_{2})$ for maximization problems.

Furthermore, $c^{I}_{1}\prec_{mw} c^{I}_{2}$ if $c^{I}_{1}\preceq_{mw} c^{I}_{2}$ and $c^{I}_{1}\neq c^{I}_{2}$.

### Definition 2

[32]

The perceived value of an interval number $c^{I}=[c^{-},c^{+}]$ is defined as:

$$o\bigl(c^{I}\bigr)=\gamma c^{-}+(1-\gamma)c^{+}, $$

where $\gamma (0\leq\gamma\leq1)$ is the optimism degree of decision makers.

### Definition 3

[28]

For any two interval numbers $c^{I}_{1}=[c^{-}_{1},c^{+}_{1}]$ and $c^{I}_{2}=[c^{-}_{2},c^{+}_{2}]$, a preference-based index can be defined as:

$$\delta\bigl(c^{I}_{1}\prec c^{I}_{2} \bigr)=\frac {o(c^{I}_{2})-o(c^{I}_{1})}{w(c^{2}_{1})+w(c^{I}_{1})+1}=\frac{(\gamma c^{-}_{2}+(1-\gamma)c^{+}_{2})-(\gamma c^{-}_{1}+(1-\gamma )c^{+}_{1})}{\frac{1}{2}(c^{+}_{2}-c^{-}_{2})+\frac {1}{2}(c^{+}_{1}-c^{-}_{1})+1}, $$

where ≺ denotes $c^{I}_{1}$ to be inferior to $c^{I}_{2}$, $o(c^{I}_{1})$ and $o(c^{I}_{2})$ denote the perceived values of $c^{I}_{1}$ and $c^{I}_{2}$, respectively. If $\delta (c^{I}_{1}\prec c^{I}_{2})>0$, $c^{I}_{1}$ is preferred to $c^{I}_{2}$ for a minimization problem.

## Interval bilevel linear programming problem

Consider the following interval bilevel linear programming problem with interval objectives and interval constraints:

1 $$ \textstyle\begin{cases} {\min_{x_{1}}} & [c^{-}_{11},c^{+}_{11}]x_{1}+[c^{-}_{12},c^{+}_{12}]x_{2}\\ & \text{where } x_{2} \text{ solves}\\ {\min_{x_{2}}} & [c^{-}_{21},c^{+}_{21}]x_{1}+[c^{-}_{22},c^{+}_{22}]x_{2}\\ \mathrm{s.t.} & [a^{-}_{i1},a^{+}_{i1}]x_{1}+[a^{-}_{i2},a^{+}_{i2}]x_{2}\geq [b^{-}_{i},b^{+}_{i}],\quad i=1,2,\ldots,m,\\ & x_{1}\geq0,x_{2}\geq0, \end{cases} $$

where $x_{1}$ and $x_{2}$ are $n_{1}$-dimensional upper level decision variable column vector and $n_{2}$-dimensional lower level decision variable column vector, respectively, $[c^{-}_{lj},c^{+}_{lj}]$, $l,j=1,2$, and $[a^{-}_{ij},a^{+}_{ij}]$, $i=1,2,\ldots,m$, are $n_{j}$-dimensional interval vectors whose components are all intervals, and $[b_{i}^{-},b_{i}^{+}]$, $i=1,2,\ldots ,m$ are interval numbers.

Here, it is important to realize that the meaning of minimizing both objective functions and inequality constraints is not clear at all owing to the presence of interval coefficients in problem (1). In other words, how to tackle the relationship between the left and the right hand sides of the interval inequality constraints and how to search the optimal values for the interval objective functions are two important issues. Thus we cannot blindly apply solution concepts and existing approaches for deterministic bilevel cases to handle this type of problem. For this purpose, we will develop a solution methodology from a new perspective to transform and deal with problem (1) in the following section.

## Solution methodology

In this section, we develop a novel approach on the basis of preference-based index to convert and cope with the interval bilevel linear programming problem (1).

### Conversion of the interval constraint region into its crisp one

To deal with problem (1), we first shall discuss crisp equivalent transformation for the interval constraint region in problem (1). In order to do so, we first need to convert each interval inequality constraint into its crisp form. From the point of decision maker’s satisfaction, several typical methods introduced by Sengupta *et al.* [33], Guo and Wu [34], and Allahdadi and Nehi [35] mainly focus on transforming an interval inequality constraint into a type of satisfactory crisp constraint. However, it has been pointed out by Chen *et al.* [27] that these types of conversion could lose the uncertainty of intervals in part during the transformation process. In a different approach, recently Chen *et al.* [27] discussed a new equivalent transformation for interval inequality constraint in terms of normal variation of interval number and chance-constrained programming approach. The main advantage of this kind of transformation is to maintain as much uncertainty as possible. In this way, we first transform any interval inequality constraint of problem (1) into a stochastic inequality form by normal variation of intervals.

For any interval $a^{I}=[a^{-},a^{+}]$, in terms of the 3*σ* law, its corresponding normally distributed random variable, denoted by $\bar {a}\sim N(\mu,\sigma^{2})$, can be determined as follows:

$$\mu=\frac{a^{-}+a^{+}}{2},\qquad \sigma=\frac{a^{+}-a^{-}}{6}. $$

Clearly, we have $[a^{-},a^{+}]=[\mu-3\sigma,\mu+3\sigma]$. Notice that the probability that any interval number *ā* falls in $[a^{-},a^{+}]$ is 99.73% on the basis of the 3*σ* law. Thus it is reasonable to utilize a normally distributed random variable to represent an interval.

By replacing interval coefficients with their corresponding normally distributed random variables, the *i*th interval inequality constraint of problem (1) can be converted as:

$$\bar{a}_{i1}x_{1}+\bar{a}_{i2}x_{2}\geq \bar{b}_{i},\quad i=1,2,\ldots,m, $$

where $\bar{a}_{ij}=(\bar{a}_{ij1},\bar{a}_{ij2},\ldots,\bar {a}_{ijn_{j}})$, $i=1,2,\ldots,m$, $j=1,2$, are corresponding normally distributed random vectors of interval vectors $[a_{ij}^{-},a_{ij}^{+}]$, and $\bar{b}_{i}$, $i=1,2,\ldots,m$ are corresponding normally distributed random variables of interval numbers $[b_{i}^{-},b_{i}^{+}]$. For simplicity, we assume that $\bar {a}_{ij1},\bar{a}_{ij2},\ldots,\bar{a}_{ijn_{j}}$ and $\bar{b}_{i}$ are independent from each other.

As is well known, chance-constrained programming approach [36] is the most applied one to handle the stochastic constraints. By this means, stochastic constraints hold at least some satisficing probability level specified by decision makers. With the aid of chance-constrained programming, the above *i*th stochastic inequality constraint can be reformulated as follows:

$$\operatorname{Pr}\{\bar{a}_{i1}x_{1}+\bar{a}_{i2}x_{2} \geq\bar{b}_{i}\}\geq \beta_{i},\quad i=1,2,\ldots,m, $$

where Pr denotes the probability of the event, and $\beta_{i}\in(0,1)$ is the probability level of the *i*th constraint, $i=1,2,\ldots,m$.

In terms of Theorem 5.1 in [27], the crisp equivalent form of the above *i*th chance inequality constraint can be obtained.

Now we have converted the interval constraint region of problem (1) into its crisp structure. Then problem (1) can be transformed into the following problem:

2 $$ \textstyle\begin{cases} {\min_{x_{1}}} & [c^{-}_{11},c^{+}_{11}]x_{1}+[c^{-}_{12},c^{+}_{12}]x_{2}\\ & \text{where } x_{2} \text{ solves}\\ {\min_{x_{2}}} & [c^{-}_{21},c^{+}_{21}]x_{1}+[c^{-}_{22},c^{+}_{22}]x_{2}\\ \mathrm{s.t.} & \sum_{s=1}^{n_{1}}E(\bar{a}_{i1s})x_{1s}\\ &\qquad {}+\sum_{t=1}^{n_{2}}E(\bar{a}_{i2t})x_{2t}\\ &\qquad {}+\Phi^{-1}(\beta_{i})\sqrt{\sum_{s=1}^{n_{1}}V(\bar{a}_{i1s})x^{2}_{1s}+\sum_{t=1}^{n_{2}}V(\bar {a}_{i2t})x^{2}_{2t}+V(\bar{b}_{i})}\\ &\quad \geq E(\bar{b}_{i}),\quad i=1,2,\ldots,m,\\ & x_{1}=(x_{11},x_{12},\ldots,x_{1n_{1}})^{\mathrm{ T}}\geq 0,x_{2}=(x_{21},x_{22},\ldots,x_{2n_{2}})^{\mathrm{ T}}\geq0, \end{cases} $$

where $E(\cdot)$ and $V(\cdot)$ denote the expectation and variance of a random variable, and $\Phi^{-1}$ is the inverse function of standardized normal distribution.

For convenience, we denote the constraint region of problem (2) by *D*.

Equivalently, problem (2) can be rewritten as

3 $$ \textstyle\begin{cases} {\min_{x_{1}}} & F^{I}(x_{1},x_{2})=[c^{-}_{11},c^{+}_{11}]x_{1}+[c^{-}_{12},c^{+}_{12}]x_{2}\\ & \text{where } x_{2} \text{ solves}\\ {\min_{x_{2}}} & f^{I}(x_{1},x_{2})=[c^{-}_{21},c^{+}_{21}]x_{1}+[c^{-}_{22},c^{+}_{22}]x_{2}\\ \mathrm{s.t.} & (x_{1},x_{2})\in D, \end{cases} $$

where $F^{I}=[F^{-},F^{+}]$ and $f^{I}=[f^{-},f^{+}]$ denote interval objective functions of the upper and lower level programming problems, respectively.

Clearly, the above problem is a bilevel programming problem with interval coefficients in both objective functions only.

### Preference-based deterministic bilevel programming problem

In order to reflect different preferences of different decision makers, in this section we first introduce the concept of the preference level that the interval objective function is preferred to a target interval in light of preference-based index in [28]. Then we build a preference-based deterministic bilevel programming problem for problem (3) based on the preference level and the order relation $\preceq_{mw}$.

For a given $x_{1}$, the lower level programming problem of problem (3) is an essentially single level optimization problem for objective function involving interval coefficients. For this type of problem, the midpoints of the intervals are often used to cope with the related interval objective functions, but it may cause the loss of helpful information to some extent in terms of such a treatment. To better reflect uncertain information, the order relation $\leq_{mw}$ which considers the midpoint and half-width of intervals at the same time is employed to compare different interval objective function values of the lower level problem for different decision variables. For given $x_{1}$, denote the feasible region of the lower level problem by $D(x_{1})$. Based on the linear combination method, then the lower level problem can be converted into the following problem:

4 $$ \textstyle\begin{cases} {\min_{x_{2}}} & \theta m(f^{I}(x_{1},x_{2}))+(1-\theta )w(f^{I}(x_{1},x_{2}))\\ & x_{2}\in D(x_{1}), \end{cases} $$

where $\theta (0\leq\theta\leq1)$ denotes a weighting factor, $m(f^{I}(x_{1},x_{2}))=\frac {f^{-}(x_{1},x_{2})+f^{+}(x_{1},x_{2})}{2}$ and $w(f^{I}(x_{1}, x_{2}))=\frac{f^{+}(x_{1},x_{2})-f^{-}(x_{1},x_{2})}{2}$ represent the midpoint and half-width of the lower level objective function $f^{I}(x_{1},x_{2})$, respectively.

Denote the set of optimal solutions of problem (4) by $M^{I}(x_{1})$ for a given $x_{1}$. Then the feasible region of problem (3) can be defined as:

$$IR^{I}=\bigl\{ (x_{1},x_{2})|(x_{1},x_{2}) \in D, x_{2}\in M^{I}(x_{1})\bigr\} . $$

Thus problem (3) can be formulated as:

5 $$ \textstyle\begin{cases} {\min_{x_{1},x_{2}}} & F^{I}(x_{1},x_{2})\\ \mathrm{s.t.} & (x_{1},x_{2})\in IR^{I}. \end{cases} $$

It is well known that the order relation between intervals is often applied to tackle interval objective function. So far there have been all sorts of definitions of the interval order relations based on various mathematical approaches [37–39] with the exception of the order relation $\preceq_{mw}$ mentioned above. Among them, Sengupta and Pal [38] proposed the acceptability index for ranking any two interval numbers based on decision maker’s satisfaction. It has been pointed out by Ruan *et al.* [28] that this index cannot be applied for reflecting different preferences from different decision makers. For this purpose, we apply preference-based index for ranking interval numbers introduced by Ruan *et al.* [28] to treat the interval objective function in the upper level of problem (5).

In order to find a satisfying solution for the decision maker at the upper level, the interval objective function often needs to satisfy a desired target interval determined in advance by the decision maker as far as possible. Let $C^{I}=[C^{-},C^{+}]$ be a predetermined target interval corresponding to the upper level objective function. Based on preference-based index for ranking interval numbers, we define the concept of the preference level as follows.

#### Definition 4

For any $(x_{1},x_{2})\geq0$, $\delta (F^{I}(x_{1},x_{2})\prec C^{I})$ is said to be a preference level that the interval objective function $F^{I}(x_{1},x_{2})$ is inferior to a target interval $C^{I}$. If $\delta(F^{I}(x_{1},x_{2})\prec C^{I})>0$, $F^{I}(x_{1},x_{2})$ is preferred to $C^{I}$ for a minimization problem.

Clearly, it needs to maximize the preference level of the interval objective function to be inferior to its target interval for a minimization problem. In this sense, problem (5) can be transformed into the following preference-based deterministic bilevel programming problem:

6 $$ \textstyle\begin{cases} {\max_{x_{1},x_{2}}} & \delta(F^{I}(x_{1},x_{2})\prec C^{I})\\ \mathrm{s.t.} & (x_{1},x_{2})\in IR^{I}. \end{cases} $$

Now we give the concept of a preference *δ*-optimal solution for the interval bilevel linear programming problem (1).

#### Definition 5

A solution $(x^{*}_{1},x^{*}_{2})\in IR^{I}$ is said to be a preference *δ*-optimal solution of problem (1) if there does not exist another feasible solution $(x_{1},x_{2})\in IR^{I}$ such that $\delta(F^{I}(x_{1},x_{2})\prec C^{I})\geq\delta (F^{I}(x^{*}_{1},x^{*}_{2})\prec C^{I})$.

Using Definition 3, we have

$$\delta\bigl(F^{I}(x_{1},x_{2})\prec C^{I}\bigr)=\frac {o(C^{I})-o(F^{I}(x_{1},x_{2}))}{w(C^{I})+w(F^{I}(x_{1},x_{2}))+1}. $$

Thus problem (6) can be rewritten as

7 $$ \textstyle\begin{cases} {\max_{x_{1}}} & \frac {o(C^{I})-o(F^{I}(x_{1},x_{2}))}{w(C^{I})+w(F^{I}(x_{1},x_{2}))+1}\\ & \text{where } x_{2} \text{ solves}\\ {\min_{x_{2}}} & \theta m(f^{I}(x_{1},x_{2}))+(1-\theta )w(f^{I}(x_{1},x_{2}))\\ & \sum_{s=1}^{n_{1}}E(\bar{a}_{i1s})x_{1s}\\ &\qquad{}+\sum_{t=1}^{n_{2}}E(\bar {a}_{i2t})x_{2t}\\ &\qquad{}+\Phi^{-1}(\beta_{i})\sqrt{\sum_{s=1}^{n_{1}}V(\bar {a}_{i1s})x^{2}_{1s}+\sum_{t=1}^{n_{2}}V(\bar {a}_{i2t})x^{2}_{2t}+V(\bar{b}_{i})}\\ & \quad\geq E(\bar{b}_{i}),\quad i=1,2,\ldots,m,\\ & x_{1}=(x_{11},x_{12},\ldots,x_{1n_{1}})^{\mathrm{ T}}\geq 0,x_{2}=(x_{21},x_{22},\ldots,x_{2n_{2}})^{\mathrm{ T}}\geq0. \end{cases} $$

On the other hand, if the original problem is a type of maximization stated as follows:

8 $$ \textstyle\begin{cases} {\max_{x_{1}}} & [c^{-}_{11},c^{+}_{11}]x_{1}+[c^{-}_{12},c^{+}_{12}]x_{2}\\ & \text{where } x_{2} \text{ solves}\\ {\max_{x_{2}}} & [c^{-}_{21},c^{+}_{21}]x_{1}+[c^{-}_{22},c^{+}_{22}]x_{2}\\ \mathrm{s.t.} & [a^{-}_{i1},a^{+}_{i1}]x_{1}+[a^{-}_{i2},a^{+}_{i2}]x_{2}\geq [b^{-}_{i},b^{+}_{i}],\quad i=1,2,\ldots,m,\\ & x_{1}\geq0,x_{2}\geq0. \end{cases} $$

For problem (8), the decision maker hopes to maximize the preference level of the interval objective function to be superior to its target interval. By using the similar idea for the above minimization, another preference-based deterministic bilevel programming problem of problem (8) can be expressed as:

9 $$ \textstyle\begin{cases} {\max_{x_{1}}} & \delta(F^{I}(x_{1},x_{2})\succ C^{I})\\ & \text{where } x_{2} \text{ solves}\\ {\max_{x_{2}}} & \theta m(f^{I}(x_{1},x_{2}))+(1-\theta )(-w(f^{I}(x_{1},x_{2})))\\ & (x_{1},x_{2})\in D. \end{cases} $$

Equivalently, problem (9) can be rewritten in the form

10 $$ \textstyle\begin{cases} {\max_{x_{1}}} & \frac {o(F^{I}(x_{1},x_{2}))-o(C^{I})}{w(F^{I}(x_{1},x_{2}))+w(C^{I})+1}\\ & \text{where } x_{2} \text{ solves}\\ {\max_{x_{2}}} & \theta m(f^{I}(x_{1},x_{2}))+(1-\theta )(-w(f^{I}(x_{1},x_{2})))\\ & \sum_{s=1}^{n_{1}}E(\bar{a}_{i1s})x_{1s}\\ &\qquad{}+\sum_{t=1}^{n_{2}}E(\bar {a}_{i2t})x_{2t}\\ &\qquad{}+\Phi^{-1}(\beta_{i})\sqrt{\sum_{s=1}^{n_{1}}V(\bar {a}_{i1s})x^{2}_{1s}+\sum_{t=1}^{n_{2}}V(\bar {a}_{i2t})x^{2}_{2t}+V(\bar{b}_{i})}\\ &\quad \geq E(\bar{b}_{i}),\quad i=1,2,\ldots,m,\\ & x_{1}=(x_{11},x_{12},\ldots,x_{1n_{1}})^{\mathrm{ T}}\geq 0,x_{2}=(x_{21},x_{22},\ldots,x_{2n_{2}})^{\mathrm{ T}}\geq0. \end{cases} $$

Clearly, problems (7) and (10) are a class of nonlinear bilevel programming problems which are coped with by one of the metaheuristic techniques namely estimation of distribution algorithm in the next subsection.

### Solution approach based on estimation of distribution algorithm

It is well known that bilevel programming problem is a complex optimization model and it is difficult to tackle. Usually, traditional solution methods involve huge computational load when solving this type of problem and they are only successful for some special bilevel cases. Estimation of distribution algorithm (EDA) [40], which is a new evolutionary metaheuristic algorithm, has attracted considerable attention as an alternative method for solving bilevel programming problem [41, 42] in recent years. For EDA, the main steps of the iterative procedure include: randomly create initial population, select some excellent individuals, build a probabilistic model based on excellent individuals chosen, generate new individuals by sampling from the constructed probabilistic model, and repeat the cycle until a stopping criterion is met. Notice that the main characteristics of this approach is to reproduce a new generation implicitly by sampling from a probability model constructed by promising candidate solutions.

In our work, estimation of distribution algorithm is applied to solve nonlinear bilevel programming problems (7) and (10). For these two problems, observing that the lower level objective functions are linear and constraint functions are quadratic, thus a number of traditional techniques can be employed to solve the lower level problem. Furthermore, estimation of distribution algorithm is used to deal with the upper level problem. Based on these ideas, we give the details of the computational method by combining estimation of distribution algorithm with some traditional method for solving problems (7) and (10) as follows:

Step 1 Ask the decision makers to specify the probability levels $\beta_{i}, i=1,2,\ldots,m$, weighting factor *θ*, target interval $C^{I}$ and optimism degree of the upper level decision maker *γ*. Generate the initial population $\mathrm{Pop}(0)$ with population size *N* comprised by the upper level decision variable. Let $t = 0$.
Step 2 For each given upper level individual, we solve the lower level problem by means of some traditional method.
Step 3 Evaluate the fitness value defined by the upper level objective function value for each individual.
Step 4 Select *M* best individuals from $\mathrm{Pop}(t)$ to form the parent set $Q(t)$ by the truncation selection, and update the probability model by estimating the distribution of $Q(t)$.
Step 5 Sample *N* new individuals from the updated probabilistic model. Denote the set of all these individuals by $O(t)$. Select *N* best offspring candidates from the set $\mathrm{Pop}(t)\cup O(t)$ to construct the next population $\mathrm{Pop}(t+1)$.
Step 6 If the algorithm is executed to the maximal number of generations, then stop; otherwise, let $t=t+1$, go to Step 2.

## Numerical examples and discussion

To show the feasibility and efficiency of the proposed approach for handling the interval bilevel linear programming problem, we test three numerical examples in this section. Furthermore, some comparisons between the proposed method and some existing approaches are made to better demonstrate advantages of the proposed approach.

### Numerical examples

The following three examples are selected from the existing literature.

#### Example 1

This example extracted from the literature [22] is an interval bilevel linear programming problem with only one interval inequality constraint:

11 $$ \textstyle\begin{cases} {\max_{x}} & [3,6]x+[4,9]y\\ & \text{where } y \text{ solves}\\ {\max_{y}} & [11,13]x+[7,9]y\\ \mathrm{s.t.} & [3, 5]x+[4,6]y\geq16,\\ & x+y\leq6,\\ & x\geq0, y\geq0. \end{cases} $$

#### Example 2

The following interval bilevel linear programming problem in which all the coefficients in constraints are interval numbers is taken from [25]:

12 $$ \textstyle\begin{cases} {\min_{x}} & [0,1]x+[-1,2]y\\ & \text{where } y \text{ solves}\\ {\min_{y}} & [1,3]y\\ \mathrm{s.t.} & [\frac{34}{35},1]x+[\frac{17}{10},2]y\geq[10,\frac {51}{5}],\\ & [-\frac{7}{6},-1]x+[\frac{7}{5},2]y\geq[-6,-\frac{161}{30}],\\ & [-\frac{5}{2},-2]x+[\frac{1}{2},1]y\geq[-21,-20],\\ & [-\frac{63}{40},-1]x+[-\frac{21}{10},-2]y\geq[-38,-\frac {1407}{40}],\\ & [\frac{7}{15},1]x+[-\frac{21}{10},-2]y\geq[-18,-\frac{84}{5}],\\ & x\geq0, y\geq0. \end{cases} $$

#### Example 3

Consider the following interval bilevel linear programming problem from [43]:

13 $$ \textstyle\begin{cases} {\min_{x}} & [-1,-0.5]y\\ & \text{where } y \text{ solves}\\ {\min_{y}} & [1,2]y\\ \mathrm{s.t.} & [0.5,1]x+[1.9,2]y\geq[10,10.5],\\ & [-2,-1]x+[1,2]y\geq[-6,-5],\\ & [-3,-2]x+[0.5,1]y\geq[-21,-20],\\ & [-2,-1]x+[-3,-2]y\geq[-38,-37],\\ & [0.5,1]x+[-3,-2]y\geq[-18,-17],\\ & x\geq0, y\geq0. \end{cases} $$

### Results and discussion

In this section, we show the computational results of three numerical examples to assess the performance of the proposed approach. The parameters, population size of each generation, size of the selected population, maximum number of generations, associated with estimation of distribution algorithm are taken as 50, 15 and 100, respectively.

In order to deal with these examples, first we give normal distribution random variables corresponding to interval coefficients of all the constraints of such problems according to the 3*σ* law. Tables 1-3 present the relations of interval coefficients and corresponding random variables with normal distributions for these three examples. Base on these results, all interval inequality constraints of these problems can be reformulated into stochastic equivalent forms. For simplicity, each chance-constrained probability related to the resulted stochastic inequality constraints is set as $\beta=0.95$, and we have $\Phi ^{-1}(\beta)=1.645$. Based on the proposed models (7) and (10), these interval bilevel linear programming problems can be converted into their equivalent deterministic ones.

**Table 1:** **Normal distributions corresponding to interval coefficients in the first constraint of Example** **1**

**Interval**	$\boldsymbol {N(\mu,\sigma^{2})}$	**Interval**	$\boldsymbol {N(\mu,\sigma ^{2})}$
[3,5]	*N*(4,0.3333^2^)	[4,6]	*N*(5,0.3333^2^)

**Table 2:** **Normal distributions corresponding to interval coefficients in all the constraints of Example** **2**

**Interval**	$\boldsymbol {N(\mu,\sigma^{2})}$	**Interval**	$\boldsymbol {N(\mu,\sigma ^{2})}$	**Interval**	$\boldsymbol {N(\mu,\sigma^{2})}$
$[\frac{34}{35},1]$	*N*(0.9857,0.0048^2^)	$[\frac{17}{10},2]$	*N*(1.85,0.05^2^)	$[10,\frac{51}{5}]$	*N*(10.1,0.0333^2^)
$[-\frac{7}{6},-1]$	*N*(−1.0833,0.0278^2^)	$[\frac{7}{5},2]$	*N*(1.7,0.1^2^)	$[-6,-\frac{161}{30}]$	*N*(−5.6833,0.1056^2^)
$[-\frac{5}{2},-2]$	*N*(−2.25,0.0833^2^)	$[\frac{1}{2},1]$	*N*(0.75,0.0833^2^)	[−21,−20]	*N*(−20.5,0.1667^2^)
$[-\frac{63}{40},-1]$	*N*(−1.2875,0.0958^2^)	$[-\frac{21}{10},-2]$	*N*(−2.05,0.0167^2^)	$[-38,-\frac{1407}{40}]$	*N*(−36.5875,0.4708^2^)
$[\frac{7}{15},1]$	*N*(0.7333,0.0889^2^)	$[-\frac{21}{10},-2]$	*N*(−2.05,0.0167^2^)	$[-18,-\frac{84}{5}]$	*N*(−17.4,0.2^2^)

**Table 3:** **Normal distributions corresponding to interval coefficients in all the constraints of Example** **3**

**Interval**	$\boldsymbol {N(\mu,\sigma^{2})}$	**Interval**	$\boldsymbol {N(\mu,\sigma ^{2})}$	**Interval**	$\boldsymbol {N(\mu,\sigma^{2})}$
[0.5,1]	*N*(0.75,0.0833^2^)	[1.9,2]	*N*(1.95,0.0167^2^)	[10,10.5]	*N*(10.25,0.0833^2^)
[−2,−1]	*N*(−1.5,0.1667^2^)	[1,2]	*N*(1.5,0.1667^2^)	[−6,−5]	*N*(−5.5,0.1667^2^)
[−3,−2]	*N*(−2.5,0.1667^2^)	[0.5,1]	*N*(0.75,0.0833^2^)	[−21,20]	*N*(−20.5,0.1667^2^)
[−2,−1]	*N*(−1.5,0.1667^2^)	[−3,−2]	*N*(−2.5,0.1667^2^)	[−38,−37]	*N*(−37.5,0.1667^2^)
[0.5,1]	*N*(0.75,0.0833^2^)	[−3,−2]	*N*(−2.5,0.1667^2^)	[−18,−17]	*N*(−17.5,0.1667^2^)

For Example 1, the interval target corresponding to the upper level objective functions are set as $C^{I}=[15,25]$, and the weighting factor for the lower level objective function is set as $\theta=0.4$. On the basis of model (10), then the equivalent deterministic form of this problem is expressed as follows:

14 $$ \textstyle\begin{cases} {\max_{x}} & \frac{[\gamma(3x+4y)+(1-\gamma)(6x+9y)]-[\gamma15+(1-\gamma)25]}{\frac {(6x+9y)-(3x+4y)}{2}+\frac{25-15}{2}+1}\\ & \text{where } y \text{ solves}\\ {\max_{y}} & 0.4[\frac{(13x+9y)+(11x+7y)}{2}]+0.6[-\frac {(13x+9y)-(11x+7y)}{2}]\\ \mathrm{s.t.} & 4x+5y+1.645\sqrt{0.3333^{2}x^{2}+0.3333^{2}y^{2}}\geq 16,\\ & x+y\leq6,\\ & x\geq0, y\geq0. \end{cases} $$

We apply estimation of distribution algorithm to solve model (14) with assumption that the decision makers specify the optimism degree $\gamma=0.6$. Then we obtain the following result: the optimal solution is $(x^{*},y^{*})=(0,6)$, the corresponding upper level objective value is $F_{0.6}^{I}=[24,54]$. Furthermore, we have $\delta (F_{0.6}^{I}\succ[15,25])=0.8095$. This indicates that the obtained interval value of the upper level objective function is superior to the interval goal.

For this example, Abass [22] gives the optimal solution as $(x^{*},y^{*})=(0,6)$ with the possibility degree level $\lambda=0.3$ by adopting the midpoints and half-widths of the interval coefficients to formulate both interval objective functions and applying the concept of the possibility degree of interval number to treat interval inequality constraints. As far as the final result is concerned, the result obtained by our approach is the same as that obtained in [22]. From the viewpoint of equivalent transformation of interval inequality constraint, our approach can ensure that the formulated stochastic constraint at the obtained optimal solution holds with probability of at least 95% based on chance-constrained programming, in other words, this constraint is violated with probability of at most 5%. While equivalent conversion based on the possibility degree of interval number in [22] holds at a small possibility degree level $\lambda=0.3$. As we know, a smaller possibility degree means a lower reliability of the interval constraint. Thus the result obtained by Abass’ approach [22] shows a relatively low reliability.

For Example 2, the interval goal is set as $C^{I}=[10,15]$ for the upper level objective function, and the weighting factor is set as $\theta=0.5$ for the lower level objective function. Then the equivalent deterministic bilevel problem can be stated as follows:

15 $$ \textstyle\begin{cases} {\max_{x}} & \frac{[\gamma10+(1-\gamma)15]-[\gamma(-y)+(1-\gamma )(x+2y)]}{\frac{15-10}{2}+\frac{(x+2y)-(-y)}{2}+1}\\ & \text{where } y \text{ solves}\\ {\min_{y}} & 0.5(\frac{y+3y}{2})+0.5(\frac{3y-y}{2})\\ \mathrm{s.t.} & 0.98571x+1.85y+1.645\sqrt {0.0048^{2}x^{2}+0.05^{2}y^{2}+0.0333^{2}}\geq10.1,\\ & -1.0833x+1.7y+1.645\sqrt {0.0278^{2}x^{2}+0.1^{2}y^{2}+0.1056^{2}}\geq-5.6833,\\ & -2.25x+0.75y+1.645\sqrt {0.0833^{2}x^{2}+0.0833^{2}y^{2}+0.1667^{2}}\geq-20.5,\\ & -1.2875x-2.05y+1.645\sqrt {0.0958^{2}x^{2}+0.01667^{2}y^{2}+0.4708^{2}}\\ &\quad \geq-36.5875,\\ & 0.7333x-2.05y+1.645\sqrt {0.0889^{2}x^{2}+0.0167^{2}y^{2}+0.2^{2}}\geq-17.4,\\ & x\geq0, y\geq0. \end{cases} $$

Next, we cope with the above problem by estimation of distribution algorithm with $\gamma=0.6$. The optimal solution is $(x^{*},y^{*})=(7.0,2.46)$ and the corresponding upper level objective value is $F^{I}_{0.6}=[-2.46,11.92]$. Also, we obtain $\delta (F_{0.6}^{I}\prec[10,15])=0.8146$. Thus $F_{0.6}^{I}$ is preferred to $C^{I}$ at preference-based index 0.8146.

By using two types of cutting plane methods developed by Ren and Wang [25], the best and worst optimal solutions of this example is finally obtained as $(16,11)$ and $(7,2)$, and the corresponding upper objective function values are $[-11,38]$ and $[-2,11]$. In the light of preference-based index, $\delta([-11,38]_{0.6}\prec[10,15])=0.4714$ and $\delta([-2,11]_{0.6}\prec[10,15])=0.88$ when $\gamma=0.6$. It is obvious that a modest optimal interval value of the upper level is offered to an optimistic decision maker.

For Example 3, the interval goal corresponding to the upper level objective function is specified as $C^{I}=[-1,1]$, and the weighting factor related to the lower level objective function is chosen as $\theta=0.5$. Then problem (11) can be transformed into the following equivalent deterministic bilevel problem by making use of model (7):

16 $$ \textstyle\begin{cases} {\max_{x}} & \frac{[\gamma(-1)+(1-\gamma)]-[\gamma(-y)+(1-\gamma )(x+2y)]}{\frac{1-(-1)}{2}+\frac{(x+2y)-(-y)}{2}+1}\\ & \text{where } y \text{ solves}\\ {\min_{y}} & 0.5(\frac{y+2y}{2})+0.5(\frac{2y-y}{2})\\ \mathrm{s.t.} & 0.75x+1.95y+1.645\sqrt {0.0833^{2}x^{2}+0.01667^{2}y^{2}+0.0417^{2}}\geq10.25\\ & -1.5x+1.5y+1.645\sqrt {0.1667^{2}x^{2}+0.1667^{2}y^{2}+0.1667^{2}}\geq-5.5\\ & -2.5x+0.75y+1.645\sqrt {0.1667^{2}x^{2}+0.0833^{2}y^{2}+0.1667^{2}}\geq-20.5\\ & -1.5x-2.5y+1.645\sqrt {0.1667^{2}x^{2}+0.16667^{2}y^{2}+0.16667^{2}}\geq-37.5\\ & 0.75x-2.5y+1.645\sqrt {0.0833^{2}x^{2}+0.1667^{2}y^{2}+0.1667^{2}}\geq-17.5\\ & x\geq0, y\geq0. \end{cases} $$

After solving the above problem by estimation of distribution algorithm at $\gamma=0.7$, we obtain its optimal solution $(x^{*},y^{*})=(12.4317,9.2434)$ and the corresponding upper level objective value $F_{0.7}^{I}=[-9.2434,-4.6217]$. Meanwhile, we get $\delta(F_{0.7}^{I}\prec[-1,1])=1.7297$. Clearly, $F_{0.7}^{I}$ is preferred to the interval goal $C^{I}$ with a higher preference-based index.

The best and worst optimal solutions to Example 3 obtained in [43] are $(16,11)$ and $(5.82,6.64)$, and corresponding upper level objective values are $[-11,-5.5]$ and $[-6.64,-3.32]$. According to preference-based index, when $\gamma=0.7$, we have $\delta ([-11, -5.5]_{0.7}\prec[-1,1])=3.5052$ and $\delta ([-6.64,-3.32]_{0.7}\prec[-1,1])=1.4328$. Clearly, our approach provides a modest optimal interval value for an optimistic decision maker of the upper level.

In order to show the influence of different optimism degrees from different decision makers, Table 4 provides the results of the upper level objective function and preference-based index under different optimism degrees. From Table 4, it is seen that $\gamma=0.9$ has great influence on the upper level objective function. Although the objective function values are unchanged when $\gamma=0.3$, 0.5 and 0.7, the corresponding preference-based indices are different. Clearly, pessimistic decision maker ($\gamma=0.3$), moderate decision maker ($\gamma=0.5$) and optimistic decision maker ($\gamma=0.7$) think that the interval value [$-9.2434,-4.6217$] is superior to the interval goal $C^{I}_{1}$ with a lower, moderate and greater preference-based index.

**Table 4:** **Objection function values and corresponding preference-based indices under different optimism degrees of Example** **3**

***γ***	$\boldsymbol {(x^{*},y^{*})}$	$\boldsymbol {F^{I}}$	$\boldsymbol {\delta^{F}}$
0.3	[12.4317,9.2434]	[−9.2434,−4.6217]	1.4865
0.5	[12.4317,9.2434]	[−9.2434,−4.6217]	1.6082
0.7	[12.4317,9.2434]	[−9.2434,−4.6217]	1.7298
0.9	[9.1441,11.0]	[−11,−5.5]	2.0316

## Conclusions

In this paper, we focus on a class of interval bilevel linear programming problems where all coefficients are interval numbers. To deal with these problems, a novel approach based on preference-based index is presented. In particular, we first transform the original problem into an interval bilevel programming problem with interval coefficients in both objective functions only by combining normal variation of interval number with chance-constrained programming. This type of conversion is able to keep the uncertainty of the original constraint region to a larger extent. Then we construct a preference-based deterministic bilevel programming problem by means of the preference level and the order relation $\preceq_{mw}$. Subsequently, we apply an estimation of distribution algorithm to deal with the deterministic nonlinear bilevel one. Finally, the experimental results show the effectiveness of the presented approach.

As future work, the proposed approach will be applicable to more realistic applications. In addition, we will extend the proposed method to deal with interval bilevel nonlinear programming problems.
